# Optimization of Look-Locker Turbo-Field Echo-Planar Imaging and Evaluation of Its Accuracy in Head and Neck 3D T_1_ Mapping

**DOI:** 10.2463/mrms.mp.2015-0057

**Published:** 2015-12-28

**Authors:** Masanori MAEHARA, Masahiko MONMA, Takeshi NITANAI, Tetsuya MATSUMOTO, Yukiko FUKUMA

**Affiliations:** 1 Department of Radiology, Nihon University School of Dentistry at Matsudo 2-870-1, Sakae-nishi, Matsudo-shi, Chiba 271-8587, Japan; 2 Department of Radiological Sciences, Ibaraki Prefectural University of Health Sciences; 3 Philips Electronics Japan Ltd.

**Keywords:** T_1_ mapping, LL-TFEPI, head and neck, Look-Locker

## Abstract

**Purpose:**

We present a sequence for T_1_ relaxation-time mapping that enables a rapid and accurate measuring. The sequence is based on the Look-Locker method by employing turbo-field echo-planar imaging (TFEPI) acquisitions and time to free relaxation after constant application of the radiofrequency (RF) pulses. We optimized the sequence, and then evaluated the accuracy of the method in imaging of head and neck.

**Materials and Methods:**

The method was implemented on a standard clinical scanner, and the accuracy of the T_1_ value was evaluated against that with the two-dimensional (2D) inversion recovery method.

**Results:**

The percentage errors of the T_1_ value, as validated by phantom imaging measurements, were 3.1% for slow-relaxing compartments (T_1_ = 2736 msec) and 1.1% for fast-relaxing compartments (T_1_ = 264.2 msec).

**Conclusion:**

We demonstrated a fast 3D sequence to obtain multiple slices, based on the Look-Locker method for T_1_ measurement, which provided a rapid and accurate way of measuring the spin-lattice relaxation time. An acquisition time of approximately 5 min was achieved for T_1_ mapping; in principle, this can provide head and neck coverage with 15 slices.

## Introduction

Several of the new magnetic resonance (MR) applications require quantitative measurement of the spin-lattice relaxation time (T_1_) in three dimensions (3Ds) with relatively short acquisition times. For example, quantitative tracer kinetic studies, in which vascular parameters such as blood volume and capillary permeability are calculated from dynamic contrast-enhanced MR data, require fast and accurate measurement of tissue T_1_ values. Before application of a tracer kinetic model, tissue enhancement following contrast agent administration must be converted into contrast agent concentration, and it can be shown that this calibration depends strongly on the pre-contrast tissue T_1_ value.^1–5^ Ideally, tracer kinetic studies should be done in 3D for complete characterization of a lesion, or to locate small lesions not apparent in pre-contrast scans, or to examine multiple lesions in one study. Moreover, tissue T_1_ measurement in the head and neck should be done in less than 5 min for studies to have clinically realistic acquisition times.

One of the major problems encountered in making accurate T_1_ maps is the long imaging time required. For good accuracy over a wide range of T_1_ values, multiple points on the T_1_ recovery curve must be sampled. If a conventional two-dimensional (2D) inversion recovery sequence is used, data acquisition for each slice can take a few hours. Several schemes have been developed for rapid T_1_ mapping in 2D in which multiple points on the recovery curve are sampled.^6^ These techniques include methods based on the work of Look-Locker,^7–19^ snapshot-fast low angle shot (snapshot-FLASH),^20–23^ a mixed sequence method,^24^ and a variable flip angle (VFA) method.^25,26^

The major advantages of using a sequence based on the Look-Locker method and employing turbo-field echo-planar imaging (TFEPI) acquisitions are that the acquisition time is short and the T_1_ relaxation behavior has been well-characterized. TFEPI combines turbo-field echo (TFE) and echo-planar imaging (EPI). A theoretical analysis has shown that if the Look-Locker technique is used, rather than the more time-consuming conventional inversion recovery method, T_1_ values can be measured quickly with no penalty to the signal-to-noise ratio of the calculated T_1_ map. Other authors have demonstrated a high degree of accuracy and precision achievable experimentally with this technique. However, it is difficult to apply the Look-Locker technique of multiple slices clinically, because this technique is a continuous application of the radiofrequency (RF) pulses used for imaging, and if inversion recovery pulse interval is same, sampling time becomes insufficient.

Here, we introduce a fast 3D technique for rapidly acquiring data of multiple slices for accurate T_1_ mapping. This technique is based on the principles of the 2D Look-Locker T_1_ measurement scheme, and employs TFEPI acquisitions and time to free relaxation after constant application of the RF pulses. The acquisition time needed for volumetric T_1_ mapping has been shortened considerably by segmenting the acquisition of the k_y_ phase encode lines. This technique is similar to that which might be obtained by modifying snapshot-FLASH based T_1_ measurement schemes for 3D. However, there is no delay between acquisition of successive volumes and T_1_ relaxation is governed by the principles introduced by Look and Locker. We optimized the sequence on the basis of the Look-Locker method, and then evaluated the accuracy of the method in imaging of head and neck.

### Theory

**Fig. 1.**
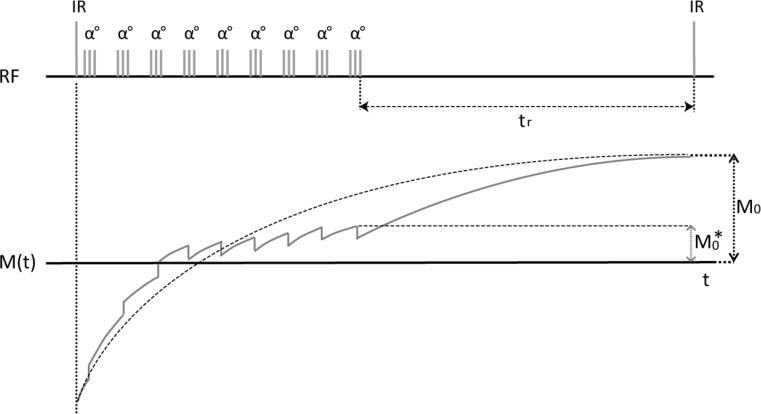
Representation of the recovery of longitudinal magnetization following an inversion pulse. This technique is based on the principles of the two-dimensional Look-Locker T_1_ measurement scheme, and employs turbo-field echo-planar imaging (TFEPI) acquisitions and time to free relaxation after constant application of the radiofrequency (RF) pulses. The relaxation process of the Look-Locker sequence is influenced by constant application of the RF pulses used for imaging after inversion pulse. The longitudinal magnetization M(t) approaches a saturation value ${\text{T}}_{1}^{*}$, which is smaller than the equilibrium value M_0_. The turbo-field echo (TFE) factor is 3 in this case; t_r_ is recovery period.

In this method, the relaxation process is influenced by constant application of the RF pulses. The effective longitudinal relaxation is determined by the effective longitudinal relaxation time ${\text{T}}_{1}^{*}$ , which is smaller than T_1_. The longitudinal magnetization M(t) approaches a saturation value, ${\text{M}}_{0}^{*}$, which is smaller than the equilibrium value M_0_ (Fig. 1). Thus, after inversion of the completely relaxed spin system, the relaxation process is described by the formula

(1) $$\text{M}(\text{t})={\text{M}}_{0}^{*}-({\text{M}}_{0}+{\text{M}}_{0}^{*})\cdot \text{exp}(-\text{t}/{\text{T}}_{1}^{*})$$

The effective longitudinal relaxation time ${\text{T}}_{1}^{*}$ is given by

(2) $$\frac{1}{{\text{T}}_{1}^{*}}=\frac{1}{{\text{T}}_{1}}-\frac{1}{\text{TR}}\cdot \text{ln}(\text{cos}\alpha )$$

where α is the flip angle.

The saturation value ${\text{M}}_{0}^{*}$ of the longitudinal magnetization is given by

(3) $${\text{M}}_{0}^{*}={\text{M}}_{0}\cdot \frac{1-\text{exp}(-\text{TR}/{\text{T}}_{1})}{1-\text{exp}(-\text{TR}/{\text{T}}_{1}^{*})}$$

This method is applicable only if the condition $\text{TR}<{\text{T}}_{1}^{*}$ holds. Thus, Eq. (3) may be simplified to

(4) $${\text{M}}_{0}^{*}={\text{M}}_{0}\cdot \frac{{\text{T}}_{1}^{*}}{{\text{T}}_{1}}$$

For the evaluation of T_1_ a three-parameter fit of the image signal intensities is performed pixelwise according to the equation

(5) $$\text{M}(\text{t})=\text{A}-\text{B}\cdot \text{exp}\left(-\frac{\text{t}}{{\text{T}}_{1}^{*}}\right)$$

Comparison with the above equations yields

(6) $$\text{A}={\text{M}}_{0}^{*}\;\;\;\;\;\;\;\;\;\;\;\;\;\text{B}={\text{M}}_{0}+{\text{M}}_{0}^{*}$$

Thus, T_1_ may be calculated directly from the fit parameters

(7) $${\text{T}}_{1}={\text{T}}_{1}^{*}\cdot \left(\frac{\text{B}}{\text{A}}-1\right)$$

The results of the pixelwise calculation can be displayed in a quantitative T_1_ map. Of note, the method does not require knowledge of the flip angle α.

## Materials and Methods

We tested the method on a phantom and a human in a 1.5-T whole-body scanner (Intera Achieva 1.5-T Nova, Philips, The Netherlands) with a maximum gradient strength of 66 mT/m and a gradient slew rate of 160 mT/m/msec using a Philips 8-channel SENSE head coil.

We measured T_1_ values using magnitude image. The maps of ${\text{T}}_{1}^{*}$, ${\text{M}}_{0}^{*}$, and M_0_ were obtained by fitting the image signal intensities on pixelwise basis to Eq. (5) using the nonlinear least-squares method by MATLAB R2014b (MathWorks, Natick, MA, USA). Moreover, T_1_ map was calculated by using Eqs. (6) and (7) with maps of ${\text{T}}_{1}^{*}$, ${\text{M}}_{0}^{*}$, and M_0._

### Phantom study

To validate the T_1_ values resulting from the sequence to be based on the Look-Locker method as described above, we applied standard inversion recovery method to a multi-compartment phantom. The phantom consists of six cylindrical sample bottles filled with water and different concentrations of gadoteridol (Gd-HP-DO3A, ProHance, Eisai Co. Ltd., Tokyo, Japan). The gadoteridol concentrations are 0.05, 0.1, 0.2, 0.5, and 1.0 mmol/L, respectively.

The 2D inversion recovery sequence is a simple acquisition with an inversion recovery pulse before an excitation pulse. The imaging parameters for acquisition of a single image are as follows and shown in Table 1: repetition time (TR) 15000 msec; echo time (TE) 20 msec; field of view (FOV) 230 × 196 mm; acquisition matrix 256 × 218; acquisition pixel size 0.9 × 0.9 mm; recon matrix 256 × 218; recon pixel size 0.9 × 0.9 mm; 1 slice with a thickness of 5 mm; band width 64.3 Hz; and sampling points at 50, 100, 200, 500, 1000, 2000, 3000, and 5000 msec. The acquisition time for this sequence is 7 h 14 min. Each measurement was performed three times, and the averaged values were used for the comparison.

To verify that we had properly chosen the EPI factor which is the number of k-space profiles collected per excitation, TFE factor which is the number of k-space profiles collected per sampling point, and recovery period (t_r_) which is time to free relaxation after constant application of the RF pulses, for the phantom measurements using the Look-Locker sequence, the experiments were repeated with all parameters held constant except for EPI factor, TFE factor, and t_r_. Three values of EPI factor (1, 3, and 11) were chosen. Because each acquisition time was the same time, three values of TFE factor (33, 11, and 3) were chosen. Furthermore, at the end of the train of α-pulses, an undisturbed recovery period was optionally inserted to allow the recovery of longitudinal magnetization before the next inversion pulse. Two values of t_r_ (3136 and 4993 msec) were chosen.

The Look-Locker sequence was used to obtain images to perform fitting of the T_1_ relaxation of the doped water in the different bottles. The measurement parameters are as follows and shown in Table 1: TR shortest (6.5, 11, and 22 msec); TE shortest (3.2, 4.8, and 11 msec); FOV 230 × 196 mm; acquisition matrix 192 × 127; acquisition pixel size 1.2 × 1.54 mm; recon matrix 256 × 218; recon pixel size 0.9 × 0.9 mm; 15 slices with a thickness of 5 mm; band width 229.2, 151.2, and 54.7 Hz; flip angle 10°; inversion recovery pulse interval 7000 msec; and sampling points is shown in Table 1. The acquisition time for this sequence is 5 min 3 sec. To demonstrate the accuracy of the T_1_ values obtained by using the Look-Locker method, we compared the resultant T_1_ values with those obtained from 2D inversion recovery method measurements and calculated the percentage errors. In each bottle a circular region of interest (ROI) comprising 15 × 15 pixels was used to calculate a mean T_1_ value.

### Volunteer study

We performed a study on a single healthy volunteer by using the 2D turbo inversion recovery and optimized 3D Look-Locker methods after obtaining their informed consent as required by our institutional review board. The 2D turbo inversion recovery sequence parameters for acquisition of a single image are as follows and shown in Table 1: TR 10000 msec; TE 20 msec; FOV 230 × 196 mm; acquisition matrix 192 × 123; acquisition pixel size 1.2 × 1.59 mm; recon matrix 256 × 218; recon pixel size 0.9 × 0.9 mm; 1 slice with a thickness of 5 mm; TSE factor 8; band width 477.8 Hz; and sampling points at 50, 100, 200, 500, 1000, 2000, and 5000 msec. The acquisition time for this sequence is 10 min 30 sec. To demonstrate the accuracy of the T_1_ values obtained by using the 2D turbo inversion recovery method, we compared the resultant T_1_ values with those obtained from 2D inversion recovery method measurements and calculated the percentage errors in the phantom study.

The 3D Look-Locker sequence parameters that we chose were those optimized in the phantom study. We compared the T_1_ values of the cerebrospinal fluid (CSF), sternocleidomastoid muscle (SCM), and parotid gland (PG) of the healthy volunteer with those obtained by using 2D turbo inversion recovery measurements and calculated the percentage errors.

## Results

**Fig. 2.**
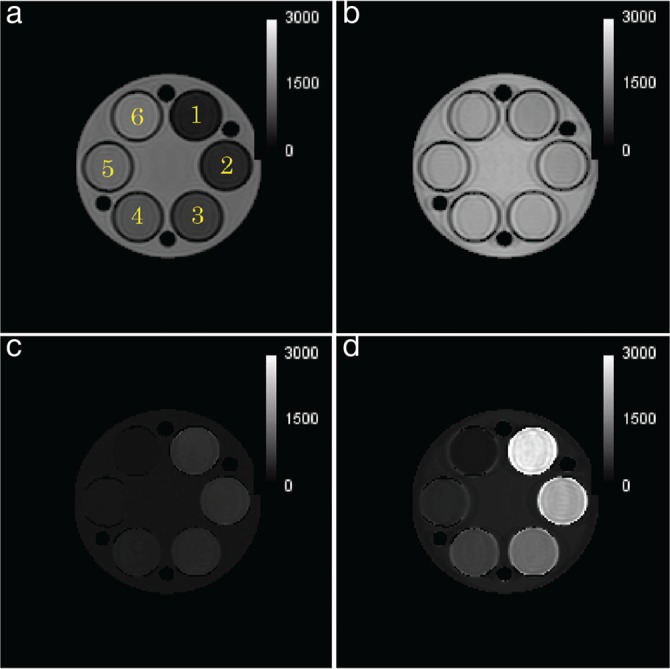
(**a**) ${\text{M}}_{0}^{*}$ map, (**b**) M_0_ map, (**c**) ${\text{T}}_{1}^{*}$ map, and (**d**) T_1_ map of a multi-compartment phantom, as generated from images acquired with the Look-Locker sequence. The locations of bottles 1, 2, 3, 4, 5, and 6 correspond to water and nominal gadoteridol concentrations of 0.05, 0.1, 0.2, 0.5, and 1.0 mmol/L, respectively. There was ghost artifact, a little.

We calculated T_1_ maps of the multi-compartment phantom by using data acquired with the Look-Locker method (Fig. 2). Tables 2 and 3 give the T_1_ values (Table 2) and percentage errors (Table 3) for the phantom measurements resulting from the 2D inversion recovery, 2D turbo inversion recovery, and 3D Look-Locker measurements. The 2D inversion recovery measurements served as a reference for all other experiments. The locations of “bottle 1,” “bottle 2,” “bottle 3,” “bottle 4,” “bottle 5,”and “bottle 6” correspond to water and the nominal gadoteridol concentrations of 0.05, 0.1, 0.2, 0.5, and 1.0 mmol/L, respectively (Fig. 2). There was good agreement between the T_1_ values from the 2D inversion recovery measurements and those from the 2D turbo inversion recovery measurements.

**Fig. 3.**
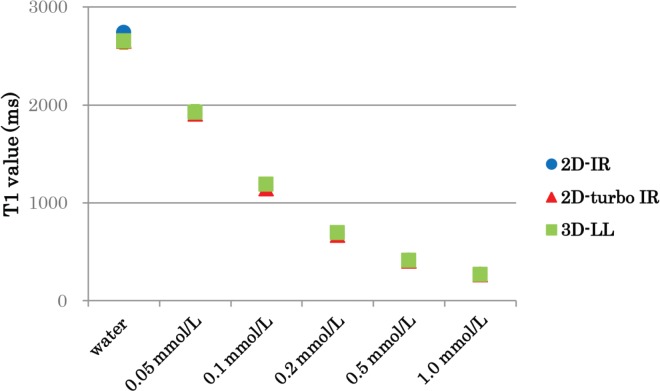
Comparison of T_1_ values of the different bottles comprising the phantom with those obtained by using the two-dimensional (2D) inversion recovery (IR) method, 2D turbo inversion recovery method, and optimized 3D Look-Locker method.

With t_r_ = 3136 msec, there was not good agreement between the 2D inversion recovery measurements and the 3D Look-Locker measurements in water and gadoteridol at the nominal concentrations of 0.05 mmol/L. With EPI factor = 1, TFE factor = 33, and EPI factor = 11, TFE factor = 3, there was not agreement in gadoteridol at the nominal concentrations of 0.5 and 1.0 mmol/L. However, with EPI factor = 3, TFE factor = 11, and t_r_ = 4993 msec, there was good agreement. These parameters gave the best agreement. We then compared the T_1_ values for the phantom measurements resulting from the 2D inversion recovery, the 2D turbo inversion recovery, and the optimized 3D Look-Locker measurements (Fig. 3). A high correlation was observed between the results obtained with the three methods.

**Fig. 4.**
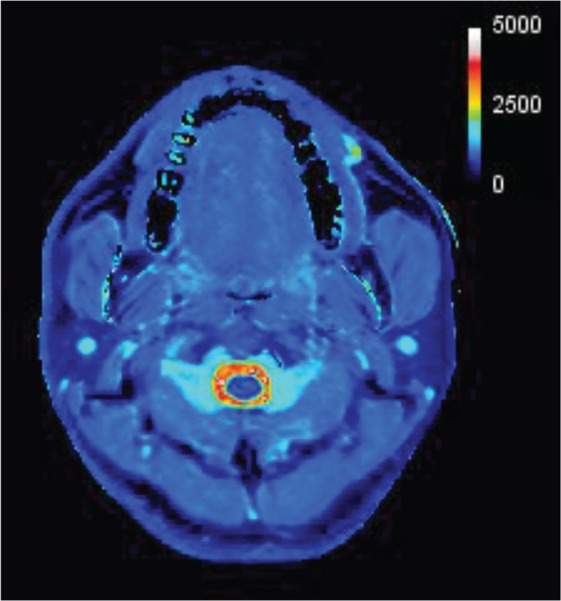
T_1_ map of a healthy volunteer, obtained by using the optimized three-dimensional Look-Locker sequence and the following parameters: repetition time (TR) 11 msec; echo time (TE) 4.8 msec; field of view (FOV) 230 × 196 mm; acquisition matrix 192 × 127; acquisition pixel size 1.2 × 1.54 mm; recon matrix 256 × 218; recon pixel size 0.9 × 0.9 mm; 15 slices with a thickness of 5 mm; echo-planar imaging (EPI) factor 3; turbo-field echo (TFE) factor 11; band width 151.2 Hz; flip angle 10°; inversion recovery (IR) pulse interval 7000 msec; recovery period (t_r_) 4993 msec; sampling points at 117 msec intervals (14, 131, 248, …, 1890 msec). The acquisition time for this sequence was 5 min 3 sec.

**Fig. 5.**
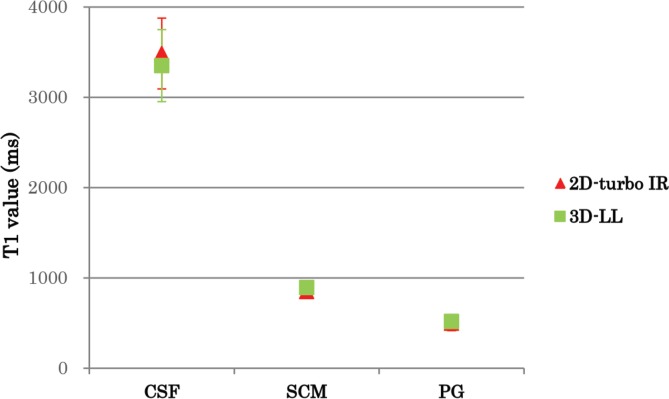
Comparison of T_1_ values of the cerebrospinal fluid (CSF), sternocleidomastoid muscle (SCM), and parotid gland (PG) of a healthy volunteer with those obtained by using the two-dimensional (2D) turbo inversion recovery method and optimized 3D Look-Locker (3D-LL) method.

We next calculated the T_1_ map of a healthy volunteer from data obtained by using the optimized 3D Look-Locker method (Fig. 4). T_1_ measurements made by using the 2D turbo inversion recovery and optimized 3D Look-Locker sequences are compared in Table 4 and Fig. 5 for ROIs in CSF, sternocleidomastoid muscle, and parotid gland. There was a good correlation between the T_1_ measurements made by using the two methods.

## Discussion

There is great interest in fast T_1_ mapping sequences, particularly for the diagnosis of different diseases,^27^ MR temperature monitoring,^28,29^ studies of intra- and extracellular water discrimination,^30^ and quantification of regional blood flow.^31^ All these measurements require highly accurate T_1_ values obtained with clinically acceptable acquisition times and with high in-planar resolution. The snapshot-FLASH sequence provides accurate and precise T_1_ mapping with high in-planar resolution and with useful acquisition times.^15^ However, for wide-range coverage of T_1_ mapping, snapshot-FLASH acquisition must be repeated, and the high in-planar resolution requirement reduces the number of time points sampled on the recovery curve. An alternative approach employs a mixed sequence to calculate images of ρ, T_1_, and T_2_ by MR imaging, although a long imaging time is required. The VFA method for 3D measurement of T_1_ values has been demonstrated by Brookes et al. This method calculates value from two spoiled gradient echo volumes acquired at two different flip angles. The advantages of the VFA method are that it is easier to implement and mapping can be calculated more rapidly, as a linear regression can be used for the fit. On the other hand, for the small TR necessary for short 3D acquisition times, it is impossible to properly optimize the choice of the two flip angles to allow accurate T_1_ measurement over a wide range of T_1_ values. When applied to 3D data, using a short TR, the VFA method suffers from poor accuracy and precision. Brookes et al. found that they could measure T_1_ values accurately only for T_1_ < 900 msec.^32^ In general, sensitivity to pulse sequence settings, such as flip angle or inversion time, is a weakness of any 2-point T_1_ measurement technique.

**Fig. 6.**
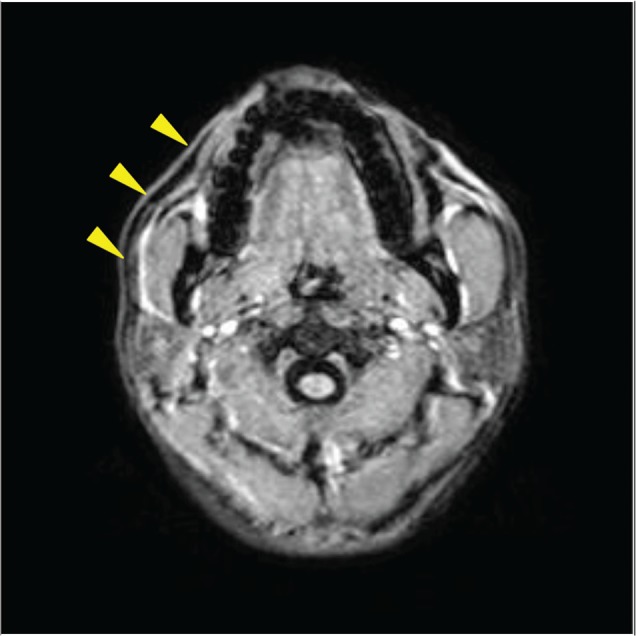
The imaging of a healthy volunteer obtained by using the optimized three-dimensional (3D) Look-Locker sequence and the following parameters: repetition time (TR) 22 msec; echo time (TE) 11 msec; field of view (FOV) 230 × 196 mm; acquisition matrix 192 × 127; acquisition pixel size 1.2 × 1.54 mm; recon matrix 256 × 218; recon pixel size 0.9 × 0.9 mm; 15 slices with a thickness of 5 mm; echo-planar imaging (EPI) factor 11; turbo-field echo (TFE) factor 3; band width 54.7 Hz; flip angle 10°; inversion recovery (IR) pulse interval 7000 msec; recovery period (t_r_) 4993 msec; sampling points at 1908 msec. Chemical shift artifact was caused significantly.

**Fig. 7.**
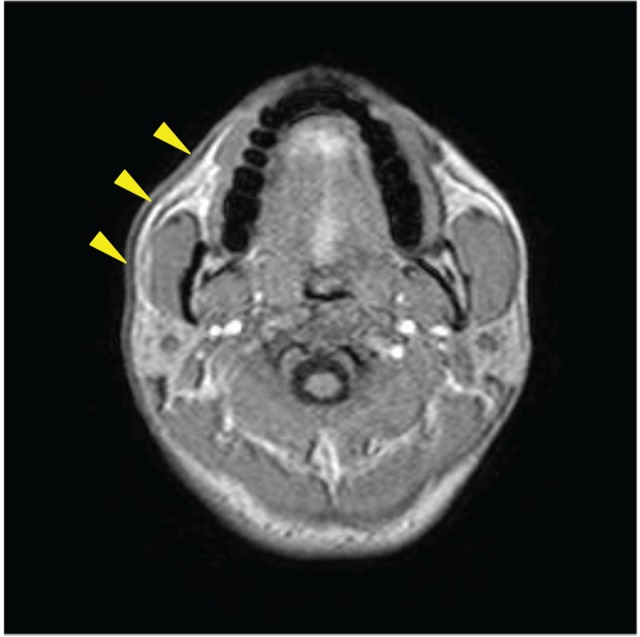
The imaging of a healthy volunteer obtained by using the optimized three-dimensional (3D) Look-Locker sequence and the following parameters: repetition time (TR) 11 msec; echo time (TE) 4.8 msec; field of view (FOV) 230 × 196 mm; acquisition matrix 192 × 127; acquisition pixel size 1.2 × 1.54 mm; recon matrix 256 × 218; recon pixel size 0.9 × 0.9 mm; 15 slices with a thickness of 5 mm; echo-planar imaging (EPI) factor 3; turbo-field echo (TFE) factor 11; band width 151.2 Hz; flip angle 10°; inversion recovery (IR) pulse interval 7000 msec; recovery period (t_r_) 4993 msec; sampling points at 1890 msec. Chemical shift artifact was caused little.

The Look-Locker sequence has made it possible to measure T_1_ values in a 3D volume in approximately 5 min with less than 3.1% error, in the case of T_1_ values between 264.2 and 2736 msec. We have found the performance of the sequence to be relatively sensitive to pulse sequence parameters. We recommend using t_r_ = 4993 msec, EPI factor = 3, and TFE factor = 11 for optimum accuracy of the T_1_ measurements. In the case of fast-relaxing compartments, the accuracy of T_1_ values declined using TFE factor = 33, because sampling intervals of data acquisitions after inversion recovery pulse are long for fast-relaxing compartments, and the accuracy declined using EPI factor = 11, because TR (22 msec) is long for short ${\text{T}}_{1}^{*}$ compartments. Moreover, EPI is highly sensitive to static magnetic field inhomogeneities, and increases chemical shift artifact (Fig. 6). This is caused by the accumulation of a phase shift. The EPI factor is one of the causes of this accumulation.^33^ In the imaging of phantom study, there was ghost artifact (Fig. 2). This is also dependent on EPI factor, but this did not have a serious problem of imaging to measure T_1_ values in the imaging of volunteer study (Fig. 4). Although, T_1_ values of parotid gland in the volunteer study were shorter than those of previous study. The reason may be that errors due to the examination of a single volunteer, imaging distortion due to the EPI factor, and changes in the signal intensity due to the phase difference between water and fat by the setting of TE had an influence on the resultant T_1_ values. In the case of slow-relaxing compartments, the accuracy declined using t_r_ = 3136 msec, because the longitudinal magnetization M(t) did not sufficiently recover to the equilibrium value M_0_. The longitudinal magnetization M(t) should recover not to the saturation value ${\text{M}}_{0}^{*}$ but to the equilibrium value M_0,_ because fitting of the recovery curve is done by using Eq. (1). In the case of optimized parameters, we thought the longitudinal magnetization M(t) of slow-relaxing compartments recovers to the equilibrium value M_0_ sufficiently, because ${\text{T}}_{1}^{*}$ value which is changed by scan parameters was very short (${\text{T}}_{1}^{*}$ value of water = 541.2 msec), and time (t_r_) to free relaxation after constant application of the RF pulses was long enough. Therefore, the accurate T_1_ mapping obtained by using the 3D Look-Locker method needs optimal sampling points and a long enough time (t_r_) to free relaxation. Moreover, the degree of local imaging distortion, chemical shift artifact, and ghost artifact is reduced sufficiently by using EPI factor = 3 in the imaging of volunteer study (Figs. 4, 7). In this study, we did not examine errors due to flip angle and RF inhomogeneities in detail because that these errors do not affect T_1_ values is implicated by the theoretical formula, and the difference of T_1_ values in each slice was less than 1.1% for bottle phantom (T_1_ = 396.6 msec). Because accuracy, scan time, and artifact are changed by scan parameters, these parameters should be chosen to suit the clinical situation.

## Conclusion

A fast 3D sequence to obtain multiple slices, based on the Look-Locker method for T_1_ measurement, provided a rapid and accurate way of measuring the spin-lattice relaxation time. The percentage errors of the T_1_ values validated by phantom imaging measurements were 3.1% for slow-relaxing compartments (water, T_1_ = 2736 msec) and 1.1% for fast-relaxing compartments (Gd-1.0 mmol/L, T_1_ = 264.2 msec). An acquisition time of approximately 5 min was achieved for T_1_ mapping; in principle, this can provide head and neck coverage with 15 slices.

## Figures and Tables

**Table 1.** The imaging parameters for acquisition of the two-dimensional (2D) inversion recovery, the 2D turbo inversion recovery, and the 3D Look-Locker sequences

2D inversion recovery sequence	
TR (msec)	15000
TE (msec)	20
FOV (mm)	230 × 196
Acquisition matrix	256 × 218
Acquisition pixel size (mm)	0.9 × 0.9
Recon matrix	256 × 218
Recon pixel size (mm)	0.9 × 0.9
Slice thickness (mm)	5
TSE factor	1
Band width (Hz)	64.3
Sampling point (msec)	50, 100, 200, 500, 1000, 2000, 5000
Scan time	7 h 14 min

2D turbo inversion recovery sequence	
TR (msec)	10000
TE (msec)	20
FOV (mm)	230 × 196
Acquisition matrix	192 × 123
Acquisition pixel size (mm)	1.2 × 1.59
Recon matrix	256 × 218
Recon pixel size (mm)	0.9 × 0.9
Slice thickness (mm)	5
TSE factor	8
Band width (Hz)	477.8
Sampling point (msec)	50, 100, 200, 500, 1000, 2000, 5000
Scan time	10 min 30 sec

3D Look-Locker sequence						
TR (msec)	6.5	11	22	6.5	11	22
TE (msec)	3.2	4.8	11	3.2	4.8	11
FOV (mm)	230 × 196					
Acquisition matrix	192 × 127					
Acquisition pixel size (mm)	1.2 × 1.54					
Recon matrix	256 × 218					
Recon pixel size (mm)	0.9 × 0.9					
Slice thickness (mm)	5					
EPI factor	1	3	11	1	3	11
TFE factor	33	11	3	33	11	3
Band width (Hz)	229.2	151.2	54.7	229.2	151.2	54.7
Flip angle (°)	10					
IR pulse interval (msec)	7000					
t_r_ (msec)	3136			4993		
Sampling point (msec)	14, 240, 467, …, 3638	14, 134, 255, …, 3744	14, 110, 206, …, 3768	14, 235, 457, …, 1786	14, 131, 248, …, 1890	14, 114, 213, …, 1908
Sampling interval (msec)	227	120	96	222	117	100
Scan time	5 min 3 sec					

EPI, echo-planar imaging; FOV, field of view; IR, inversion recovery; TE: echo time; TFE, turbo- field echo; TR, repetition time; t_r_, recovery period

**Table 2.** Comparison of calculated T_1_ values (ms) resulting from application of the two-dimensional (2D) inversion recovery method, the 2D turbo inversion recovery method, and the 3D Look-Locker method for the different bottles comprising the phantom shown in Fig. 2. Values are means ± standard deviations of T_1_ values in a homogeneous region of interest

2D inversion recovery method	
	T_1_ (ms)

Water	2736.3 ± 13.1
Gd_0.05 mmol/L	1904.8 ± 7.6
Gd_0.1 mmol/L	1157.6 ± 4.3
Gd_0.2 mmol/L	672.9 ± 1.9
Gd_0.5 mmol/L	408.1 ± 1.0
Gd_1.0 mmol/L	264.2 ± 0.8

2D turbo inversion recovery method	
	T_1_ (ms)

Water	2652.4 ± 39.1
Gd_0.05 mmol/L	1906.6 ± 21.5
Gd_0.1 mmol/L	1143.2 ± 9.0
Gd_0.2 mmol/L	670.4 ± 4.5
Gd_0.5 mmol/L	402.8 ± 1.7
Gd_1.0 mmol/L	265.1 ± 2.3

3D Look-Locker method				

		T_1_ (ms)		

		EPI factor 1	EPI factor 3	EPI factor 11
		TFE factor 33	TFE factor 11	TFE factor 3

tr_3136 ms	Water	2240.0 ± 115.8	2156.0 ± 75.4	2466.9 ± 154.1
	Gd_0.05 mmol/L	1580.5 ± 41.5	1677.9 ± 103.4	1676.5 ± 57.7
	Gd_0.1 mmol/L	1117.2 ± 21.7	1143.3 ± 27.8	1140.5 ± 28.5
	Gd_0.2 mmol/L	703.9 ± 13.9	686.2 ± 14.2	637.6 ± 13.2
	Gd_0.5 mmol/L	442.1 ± 6.5	410.0 ± 9.5	365.2 ± 9.2
	Gd_1.0 mmol/L	307.1 ± 4.9	271.3 ± 4.5	226.4 ± 7.1
tr_4993 ms	Water	2822.0 ± 210.6	2652.2 ± 81.8	2803.2 ± 231.1
	Gd_0.05 mmol/L	1821.2 ± 88.9	1922.1 ± 79.1	1807.0 ± 66.7
	Gd_0.1 mmol/L	1170.0 ± 28.2	1187.5 ± 22.5	1181.2 ± 37.7
	Gd_0.2 mmol/L	706.0 ± 9.4	691.1 ± 12.5	631.3 ± 13.5
	Gd_0.5 mmol/L	453.5 ± 6.9	411.5 ± 8.0	375.5 ± 12.0
	Gd_1.0 mmol/L	308.7 ± 4.5	267.2 ± 4.5	226.6 ± 6.0

EPI, echo-planar imaging; TFE, turbo-field echo; t_r_, recovery period

**Table 3.** Comparison of percentage errors of T_1_ values obtained from by using the two-dimensional (2D) inversion recovery method, the 2D turbo inversion recovery method, and the 3D Look-Locker method. The 2D inversion recovery measurements served as a reference. Values are percentage errors of T_1_ values in a homogeneous region of interest

2D turbo inversion recovery method	

	Percentage error (%)

Water	3.1
Gd_0.05 mmol/L	0.1
Gd_0.1 mmol/L	1.2
Gd_0.2 mmol/L	0.4
Gd_0.5 mmol/L	1.3
Gd_1.0 mmol/L	0.3

3D Look-Locker method				

		Percentage error (%)		

		EPI factor 1	EPI factor 3	EPI factor 11
		TFE factor 33	TFE factor 11	TFE factor 3

tr_3136 ms	Water	18.1	21.2	9.8
	Gd_0.05 mmol/L	17.0	11.9	12.0
	Gd_0.1 mmol/L	3.5	1.2	1.5
	Gd_0.2 mmol/L	4.6	2.0	5.2
	Gd_0.5 mmol/L	8.3	0.5	10.5
	Gd_1.0 mmol/L	16.3	2.7	14.3

tr_4993 ms	Water	3.1	3.1	2.4
	Gd_0.05 mmol/L	4.4	0.9	5.1
	Gd_0.1 mmol/L	1.1	2.6	2.0
	Gd_0.2 mmol/L	4.9	2.7	6.2
	Gd_0.5 mmol/L	11.1	0.8	8.0
	Gd_1.0 mmol/L	16.9	1.1	14.2

EPI, echo-planar imaging; TFE, turbo-field echo; t_r_, recovery period

**Table 4.** Comparison of calculated T_1_ values (ms) and their percentage errors obtained by using the two-dimensional (2D) turbo inversion recovery method and optimized 3D Look-Locker (3D-LL) method in a healthy volunteer. Values are means ± standard deviations of T_1_ values and percentage errors in a homogeneous region of interest

T_1_ (ms)			Percentage error (%)

	2D-turbo IR	3D-LL	

CSF	3484.5 ± 392.3	3350.8 ± 399.1	3.8
SCM	848.0 ± 72.1	894.0 ± 64.5	5.4
PG	497.3 ± 80.5	518.7 ± 74.3	4.3

CSF, cerebrospinal fluid; IR, inversion recovery; SCM, sternocleidomastoid muscle; PG, parotid gland
